# Near-Complete Genome Sequences of 12 Peruvian Strains of Infectious Hypodermal and Hematopoietic Necrosis Virus Infecting the Shrimp Penaeus vannamei

**DOI:** 10.1128/MRA.00169-21

**Published:** 2021-05-20

**Authors:** Luis A. Salcedo-Mejía, Yerson Durán-Ramirez, Rodolfo Z. Velazco-Peña, Joseph A. Pinto, Alfredo Rebaza-Caballero

**Affiliations:** a Laboratorio de Sanidad Acuícola-Sede Tumbes, Organismo Nacional de Sanidad Acuícola (SANIPES), Tumbes, Perú; KU Leuven

## Abstract

Infectious hypodermal and hematopoietic necrosis virus (IHHNV) is a shrimp virus listed by the World Organisation for Animal Health (OIE). In this study, we report the genomic sequences of 12 IHHNV strains obtained from shrimp samples from aquaculture cultures from the Tumbes region of Peru.

## Abstract (precis)

Infectious hypodermal and hematopoietic necrosis virus (IHHNV) is a shrimp virus, and it is listed in the World Organisation for Animal Health (OIE). In this study, we report the genomic sequences of 12 IHHNV strains obtained from shrimp samples from aquaculture cultures from the Tumbes region of Peru.

## ANNOUNCEMENT

Penaeus vannamei is the shrimp with the highest commercial value, and therefore it is the most widely cultivated species around the world, including Peru (1, 2).

Infectious hypodermal and hematopoietic necrosis virus (IHHNV; genus, *Penstyldensovirus*; subfamily, *Densovirinae*; family, *Parvoviridae*) is one of the smallest penaeid shrimp viruses (3). This virus was first reported in the 1980s in Hawaii (4, 5). Phylogenetic analysis revealed the introduction of this virus to the American continent in the 1970s (6). Interestingly, IHHNV causes high mortality in Penstyldensovirus stylirostris; however, in *P. vannamei* and *P. monodon*, mortality has not been reported. In these species, IHHNV causes slow growth and deformities in the exoskeleton (called Runt deformity syndrome) (7).

IHHNV is a 22-nm parvovirus with a nonenveloped icosahedral capsid; it has a genome of ≈3.9 kb of single-stranded DNA encoding 3 open reading frames (ORFs). IHHNV infection is considered benign and of low risk, but recent studies in *P. monodon* show that high viral loads can produce a significant economic impact (8, 9).

Sequences from Peruvian IHHNV strains have not been described. In this work, we report 12 partial genomic sequences of IHHNV strains detected in the Aquaculture Health Laboratory of the National Fisheries Health Agency (SANIPES), from shrimp from different productive zones and natural environments of the Tumbes region in Peru.

**FIG 1:**
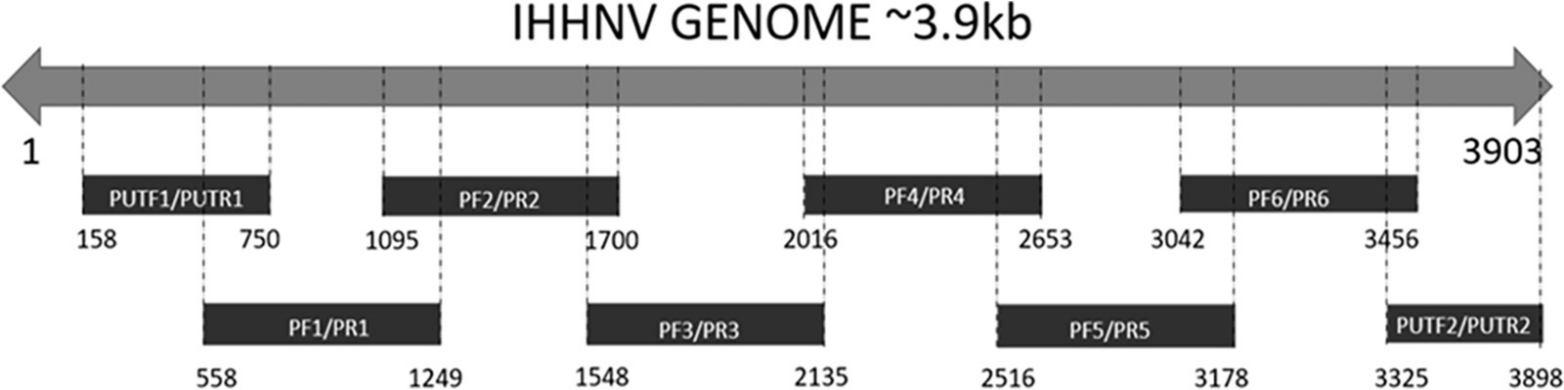
Location of the oligonucleotides in the IHHNV genome.

The sampling area included culture systems (9 samples) and natural areas (3 samples) from the north, center, and south of the coast of the Tumbes Department. DNA was isolated from 25 mg of homogenized pleopod tissue from *P. vannamei* using the DNeasy blood and tissue kit (Qiagen, Germany). The DNA was quantified with the Qubit 4 fluorometer (Invitrogen, USA). The samples were screened by endpoint PCR using the primers 309F/R, following the recommendations described in the Manual of Diagnostic Tests for Aquatic Animals from the World Organisation for Animal Health (OIE) (Fig. 1) (10).

Subsequently, to cover the IHHNV genome, 8 endpoint PCRs were performed per sample with specific primers to cover 3.9 kb using 50 to 100 ng of DNA (Fig. 1), following the protocols of Silva et al. (2014), with modifications to the amplification time (11). The PCRs were performed with high-fidelity *Taq* platinum (Thermo Scientific, USA). Sequencing was carried out using dideoxy-chain termination sequencing with the fluorescent nucleotides method. The sequences were assembled using Geneious Prime 2020.0.5 software (Biomatters, Inc.), generating a consensus sequence of ≈3.7 kb. The base calling was done with a quality score of 99.9% accuracy (Q > 30). The sequencing covered 96% of the genome (compared to the reference Hawaii IHHNV strain; GenBank accession number AF218266.2), containing 3 complete ORFs. In total, 186 nucleotides corresponding to the 5′ and 3′ untranslated regions (UTRs) were not covered in the analysis.

This draft assembly described the sequences of IHHNV strains isolated in Peru. Further investigation should be conducted to evaluate the pathogenicity of the strains present on the coast of Tumbes, Peru.

### Data availability.

The assembled genomic sequences were deposited in GenBank under accession numbers MW357689, MW357690, MW357691, MW357692, MW357693, MW357694, MW357695, MW357696, MW357697, MW357698, MW357699, and MW357700.

## References

[1] FAO. 2020. The state of world fisheries and aquaculture 2020. FAO, Rome, Italy.

[2] Ministerio de Comercio Exterior y Turismo. 2020. Reportes de comercio—reporte mensual de comercio exterior—diciembre 2019. https://cdn.www.gob.pe/uploads/document/file/533510/RMC_Diciembre_2019.pdf.

[3] Dhar AK, Cruz-Flores R, Caro LFA, Siewiora HM, Jory D. 2019. Diversity of single-stranded DNA containing viruses in shrimp. Virusdisease 30:43–57. doi:10.1007/s13337-019-00528-3. 31143831 PMC6517454

[4] Lightner DV, Redman RM, Bell TA. 1983. Infectious hypodermal and hematopoietic necrosis, a newly recognized virus disease of penaeid shrimp. J Invertebr Pathol 42:62–70. doi:10.1016/0022-2011(83)90202-1. 6886467

[5] Lightner DV, Redman RM, Bell TA, Brock JA. 1983. Detection of IHHN virus in Penaeus stylirostris and P. vannamei imported into Hawaii. J World Maric Soc 14:212–225. doi:10.1111/j.1749-7345.1983.tb00077.x.

[6] Tang KFJ, Poulos BT, Wang J, Redman RM, Shih H-H, Lightner DV. 2003. Geographic variations among infectious hypodermal and hematopoietic necrosis virus (IHHNV) isolates and characteristics of their infection. Dis Aquat Org 53:91–99. doi:10.3354/dao053091. 12650241

[7] Chai C, Liu Y, Xia X, Wang H, Pan Y, Yan S, Wang Y. 2016. Prevalence and genomic analysis of infectious hypodermal and hematopoietic necrosis virus (IHHNV) in Litopenaeus vannamei shrimp farmed in Shanghai, China. Arch Virol 161:3189–3201. doi:10.1007/s00705-016-3022-5. 27568013

[8] Tang KFJ, Lightner DV. 2006. Infectious hypodermal and hematopoietic necrosis virus (IHHNV)-related sequences in the genome of the black tiger prawn Penaeus monodon from Africa and Australia. Virus Res 118:185–191. doi:10.1016/j.virusres.2006.01.003. 16473428

[9] Sellars MJ, Cowley JA, Musson D, Rao M, Menzies ML, Coman GJ, Murphy BS. 2019. Reduced growth performance of black tiger shrimp (Penaeus monodon) infected with infectious hypodermal and hematopoietic necrosis virus. Aquaculture 499:160–166. doi:10.1016/j.aquaculture.2018.09.032.

[10] OIE. 2019. Manual of diagnostic tests for aquatic animals. OIE, Paris, France.

[11] Silva DCD, Nunes ARD, Teixeira DIA, Lima JPMS, Lanza DCF. 2014. Infectious hypodermal and hematopoietic necrosis virus from Brazil: sequencing, comparative analysis and PCR detection. Virus Res 189:136–146. doi:10.1016/j.virusres.2014.05.008. 24867614

